# The effects of a constructed closure of the Bering Strait on AMOC tipping behavior

**DOI:** 10.1126/sciadv.aeb7887

**Published:** 2026-04-24

**Authors:** Jelle Soons, Henk A. Dijkstra

**Affiliations:** Institute for Marine and Atmospheric Research Utrecht, Utrecht University, Princetonplein 5, Utrecht 3584 CC, Netherlands.

## Abstract

The Atlantic Meridional Overturning Circulation (AMOC) is a major tipping element in the present-day climate and could potentially collapse under sufficient freshwater or CO_2_ forcing. While the effect of the Bering Strait on AMOC stability has been well studied, it is unknown whether a constructed closure of this Strait can prevent an AMOC collapse under climate change. Here, we show in an Earth system Model of Intermediate Complexity that an artificial closure of the Strait can extend the safe carbon budget of the AMOC, provided that the AMOC is strong enough at the closure time. Specifically, an equilibrium AMOC under a sufficiently low additional freshwater flux has an increased safe carbon budget given a timely closure of the Strait, while for higher freshwater fluxes (and corresponding weaker AMOC), a closure reduces this budget. This indicates that constructing this closure could be a feasible climate intervention strategy to prevent an AMOC collapse.

## Teaser

A dam to close off the Bering Strait could prevent the collapse of the large-scale ocean circulation in the Atlantic.

## INTRODUCTION

The Atlantic Meridional Overturning Circulation (AMOC) is of paramount importance in regulating Earth’s climate. It transports warm surface waters from the tropics northward, which is a major reason for the relatively mild climate in Europe despite its high latitude (*1*, *2*). A key driving factor in this overturning circulation is the water mass transformation in the Nordic Seas, where the relatively warm and salty surface waters are cooled by the atmosphere and become the cold and salty North Atlantic Deep Water. This dense water mass sinks and returns southward (*3*). There is a growing concern that, under global warming, the AMOC could weaken or even shut down (*4*–*8*), with some studies even warning for an oncoming collapse this century (*9*, *10*) while others have ruled out this possibility (*11*, *12*). A possible collapse would have a major impact on the global climate, particularly Europe’s (*13*–*15*), and could be practically irreversible as multiple equilibrium states have been found consistently throughout the model hierarchy (*16*–*20*).

A non-negligible effect on the AMOC’s stability is the Bering Strait throughflow (BST). Almost 1 sverdrup of North Pacific surface water flows northward through the Bering Strait, where it eventually ends up in the Labrador and Greenland Sea joining the lower limb of the AMOC (*21*). This water is relatively fresh (~32.5 g/kg) as it originates from Antarctic Intermediate Water, and the net northward freshwater transport is roughly 80 microsverdrup (*22*). Consequently, it inhibits the northern deep water formation and, in turn, weakens the AMOC. Paleoclimate model simulations show that a closure of the Bering Strait (CBS) leads to a stronger AMOC with increased meridional heat transport (*23*–*26*). Not only because it prevents fresh North Pacific waters from entering but also because it reduces upper ocean water exchange between the Arctic and North Atlantic which also reduces the input of fresh water into the North Atlantic (*23*, *25*). Under present-day climate conditions, a closure would increase the AMOC strength by 2.5 ± 0.5 sverdrup (*24*).

Although the BST plays only a minor role in an AMOC collapse, it can considerably affect AMOC stability under freshwater flux forcing, also called hosing (*27*, *28*). Climate model simulations in (*27*) indicate that, for low hosing values, the AMOC strength is higher under CBS, but as the hosing increases, the AMOC declines more rapidly than under an open Bering Strait (OBS), resulting in the AMOC’s critical hosing value being lower for a CBS. As the AMOC decreases, the sea level in the Arctic increases, which causes the BST to decrease or even reverse (*14*, *29*). This produces an export of the added freshwater to the Pacific. Hence, the BST functions as a stabilizing mechanism for an active AMOC and as a destabilizing mechanism for a collapsed AMOC state (*24*, *27*, *30*, *31*). This stabilizing effect of the BST on a freshwater-forced AMOC is not as pronounced under CO_2_ forcing due to additional changes in the hydrological cycle (*32*). Under CO_2_ forcing, the AMOC weakens because of increased heating instead of freshening of the North Atlantic’s surface.

**Fig. 1.**
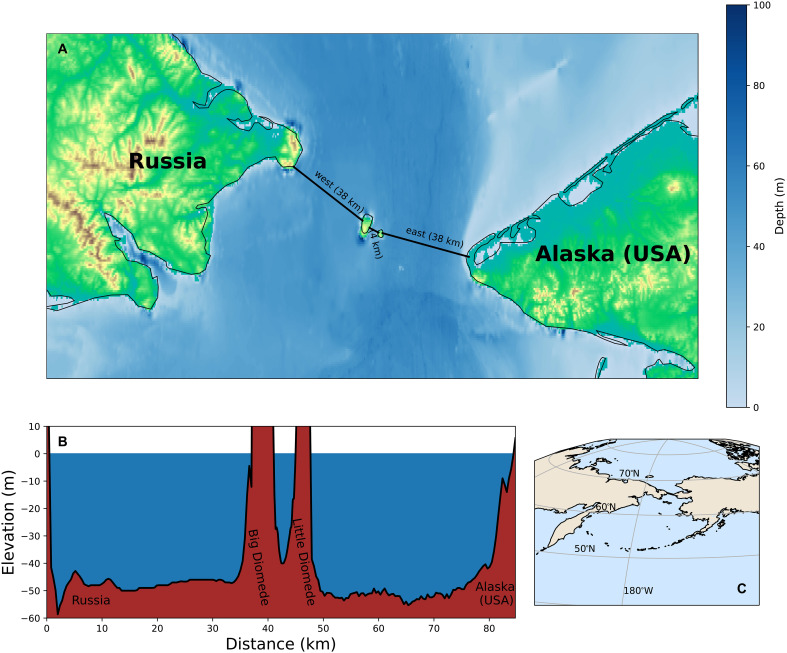
The BSD. The proposed BSD (black lines) consisting of three separate dams: a western section connecting mainland Russia with Big Diomede Island (~38 km), a middle section connecting Big Diomede Island to Little Diomede Island (~4 km), and an eastern section connecting Little Diomede Island to Alaska, USA (~38 km) (**A**), with corresponding depth profile along these transects (**B**), and the BSD in a regional overview showing it severing the Arctic-Pacific connection (**C**). Bathymetry data from (*62*).

Observation-based early warning signals are indicating that the AMOC loses resilience (*8*) and that an onset of an AMOC collapse may occur over the next decades (*9*, *10*). The consequences are substantial (*15*, *33*) and strongly motivate to consider feasible intervention strategies to prevent such a AMOC collapse. Both carbon dioxide removal techniques and solar radiation management can be effective tools to limit global warming and prevent an AMOC collapse, but their deployment comes with considerable technical, economical, and governance considerations (*34*–*38*). We here propose as an intervention the construction of a Bering Strait Dam (BSD). The BSD would disconnect the Pacific Ocean from the Arctic Ocean with three separate dams (Fig. 1). It consists of a western section connecting mainland Russia to Big Diomede Island, a middle section connecting the Diomede Islands, and an eastern section connecting Little Diomede Island to Alaska, USA. Combined, these sections have a length of roughly 80 km and encounter an average depth of 50 m with a maximum depth of 59 m (*39*, *40*). Given these dimensions, the construction of the BSD is considered to be technically feasible (*41*).

## RESULTS

We study the AMOC’s response to CO_2_ forcing and the influence of a Bering Strait closure on this response using the Earth system model CLIMBER-X (see Materials and Methods) (*42*, *43*). All components of the climate model have a horizontal resolution of 5° × 5°. Earlier work has shown that there are four distinct AMOC equilibrium states in CLIMBER-X: a strong AMOC state, a modern AMOC state, a weak AMOC state, and a collapsed AMOC (*44*). We will refer to the first two both as an ON state and the latter two as an OFF state. Note that, in this model, the produced AMOC ON state is slightly too shallow and lacks a deep southern overturning cell, while the OFF state lacks a strong reversed circulation near the surface (see also fig. S1) (*14*, *19*, *42*). At this relatively low resolution, we can simulate almost 10,000 model years per day, allowing us to do many simulations to find the safe carbon budget under different background conditions. To understand the stability of the AMOC under CO_2_ forcing, we need to consider first its sensitivity to freshwater forcing.

### Freshwater forcing

To determine the stability of the AMOC in CLIMBER-X under OBS and CBS under freshwater flux forcing, we perform two quasi-equilibrium hysteresis experiments. Here, a slowly varying freshwater flux forcing with strength $F_{H}$ is applied between latitudes 20°N and 50°N in the Atlantic. This region is a common choice in AMOC hysteresis experiments (*17*, *19*, *27*) and yields a straightforward hysteresis curve (*44*, *45*). The freshwater flux is compensated over the rest of the ocean domain. The initial state is an equilibrium AMOC state under preindustrial conditions, i.e., no hosing ($F_{H}=0$ sverdrup) and the CO_2_ concentration is fixed at 280 parts per million (ppm). The freshwater flux $F_{H}$ is increased linearly in time at a rate of 0.025 sverdrup/thousand years (kyr) until a total flux of 0.35 sverdrup is achieved, after which the flux is reduced with the same rate till the hosing flux is −0.25 sverdrup. Then, last, the hosing flux is increased to its original zero level. This rate was based on those used for quasi-equilibrium simulations in previous studies on AMOC hysteresis curves using freshwater hosing in CLIMBER-X (*44*, *45*).

**Fig. 2.**
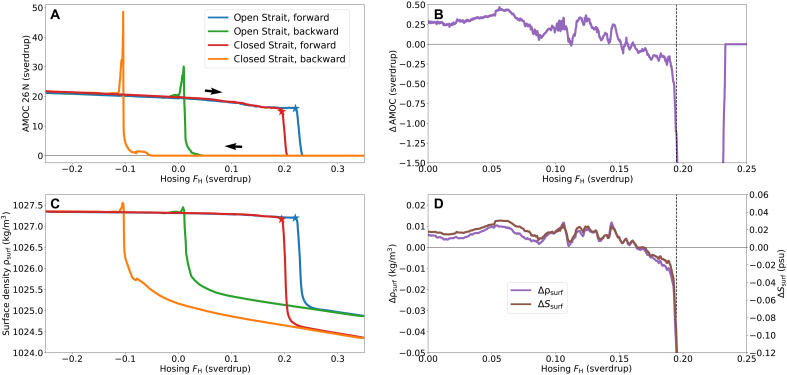
The hysteresis experiment. The quasi-equilibrium simulations for an open Strait (blue, green) and a closed Strait (red, orange), consisting of simulations where the hosing flux $F_{H}$ increases (blue, red) and decreases (green, orange). The asterisks mark the estimated tipping points of the AMOC collapses (**A** and **C**), while the vertical line (dashed, black) indicates the critical hosing value for the AMOC tipping under CBS (**B** and **D**). (A) The AMOC strength—computed as the maximum overturning strength at 26°N—for varying hosing flux $F_{H}$, where the arrows indicate the direction of time during the hosing experiment. The difference in AMOC strength (ΔAMOC) between the ON states under CBS and OBS is shown in (B). (C) The average density $\rho_{\text{surf}}$ of the top 200-m surface layer of the North Atlantic region between 50°N and 75°N and the difference in average density $\Delta \rho_{\text{surf}}$ (purple) and average salinity $\Delta S_{\text{surf}}$ (brown) in this layer between the ON states under CBS and OBS (D).

Throughout this study, we use the notation ΔQ to indicate the difference in quantity *Q* between the CBS and OBS conditions, i.e., $\Delta Q=Q_{\text{CBS}}-Q_{\text{OBS}}$. The AMOC strength at 26°N under CBS is stronger up to a hosing value $F_{H}=0.161$ sverdrup with an overturning strength of 19.9 sverdrup versus 19.6 sverdrup (CBS versus OBS) for $F_{H}=0$ sverdrup (Fig. 2, A and B). This difference is an order of magnitude smaller compared to previous studies using more detailed climate models (*23*, *24*). With the closure, the critical hosing value or an AMOC collapse is lowered to 0.195 sverdrup as opposed to 0.220 sverdrup under OBS. Moreover, the AMOC OFF state is much more stable under CBS, as its recovery occurs for a hosing value that is roughly 0.13 sverdrup lower. This also produces the larger overshoot during this recovery under CBS as a larger amount of salinity is suddenly transported northward.

The difference in surface density and salinity is rather subtle for the ON states, with initially a higher density and salinity under CBS for lower hosing values (Fig. 2, C and D). Here, the surface layer of the North Atlantic is the top 200-m layer between 50° and 75°N in the Atlantic basin (see also Materials and Methods and fig. S8). As the hosing flux increases, the difference switches sign, with both the density and salinity under CBS dipping below those under OBS, starting at $F_{H}=0.164$ sverdrup. For the OFF states, a closure results in a much lower surface density and salinity. This explains the increased stability of the AMOC OFF state under closure. All in all, a closure does increase the surface density of the North Atlantic—and correspondingly the AMOC strength—for low freshwater forcing but has the reversed effect for higher freshwater forcing and so results in a lower critical hosing value. As the difference in density is mainly explained by the difference in salinity (Fig. 2D), the difference in AMOC behavior is caused here by a difference in freshwater transports into the North Atlantic region.

**Fig. 3.**
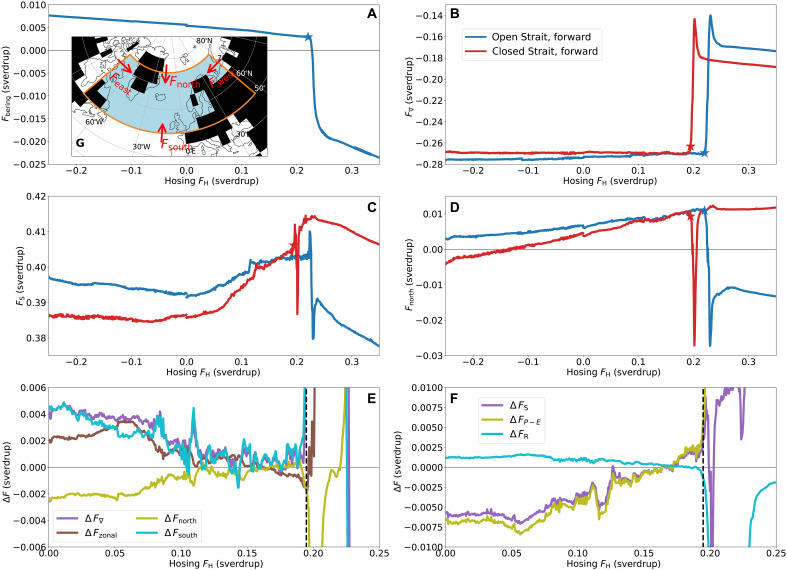
The freshwater transports. Freshwater transports for the quasi-equilibrium simulations for an open Strait (blue) and a closed Strait (red), consisting of simulations where the hosing flux $F_{H}$ increases (**A** to **D**). The asterisks mark the estimated tipping points of the AMOC collapses (A to D), while the vertical line (dashed, black) indicates the critical hosing value for the AMOC tipping under CBS (**E** and **F**). (A to D) The freshwater transports through, respectively, the Bering Strait ($F_{\text{bering}}$), the lateral boundaries of the North Atlantic region ($F_{\nabla }$), the surface of the North Atlantic region ($F_{S}$), and the northern boundary of the North Atlantic region ($F_{\text{north}}$). (E) The difference between the ON states under CBS and OBS in the freshwater transports through the North Atlantic’s boundaries ($\Delta F_{\nabla }$, purple), its zonal boundaries ($\Delta F_{\text{zonal}}$, brown), its northern boundary ($\Delta F_{\text{north}}$, citrus), and its southern boundary ($\Delta F_{\text{south}}$, cyan). (F) The difference between the ON states under CBS and OBS in the freshwater transports through the North Atlantic’s surface ($\Delta F_{S}$, purple), consisting mainly of the differences in precipitation-minus-evaporation ($\Delta F_{P-E}$, citrus) and in runoff ($\Delta F_{R}$, cyan). Inset (**G**) indicates the lateral freshwater transports and their direction (red arrows) into the North Atlantic region (light-blue, enclosed in orange). The black cells indicate grid cells with a zero ocean fraction on top of the current coastlines (black, solid).

As it is important to understand the AMOC stability under CO_2_ forcing below, we consider these freshwater transports (see Materials and Methods) in more detail for the forward runs (i.e., the simulations with slowly increasing hosing) over the North Atlantic region shown in fig. S8. Those for the backward runs are discussed in the Supplementary Materials. Note that all freshwater transports are positive when directed into the region (see also Fig. 3G). For OBS, the freshwater transport out of the North Pacific into the Arctic ($F_{\text{bering}}$ in Fig. 3A) reduces as the AMOC weakens from 5.4 to 2.9 microsverdrup at the tipping point and reverses for a collapsed AMOC to a net southward freshwater transport as high as 18.4 microsverdrup. This is qualitatively consistent with previous studies (*24*, *25*, *27*, *31*) and theory (*29*, *30*), although it is an order of magnitude smaller than current observations (*22*).

The effect of the weakening AMOC on the freshwater exchange to the North Atlantic from the rest of the ocean basin ($F_{\nabla }$), from the atmosphere, lithosphere, and cryosphere ($F_{S}$), and from the Arctic Ocean ($F_{\text{north}}$) can be seen in Fig. 3 (B to D), respectively. Under an active AMOC, there is a net freshwater export out of the North Atlantic through its lateral boundaries ($F_{\nabla }<0$; Fig. 3B) which is slightly larger for OBS. Under a collapsed AMOC, this net freshwater export has decreased and is considerably smaller under OBS. On the other hand, with a collapsed AMOC, the freshwater transport from the Arctic to the North Atlantic is southward under CBS (Fig. 3D) while northward under OBS, explaining the much fresher North Atlantic under CBS and hence the more stable OFF state. The difference $\Delta F_{\text{north}}$ (green curve in Fig. 3E) for an AMOC ON state is much more subtle, with a slightly larger southward freshwater transport under OBS, before the difference is negligible for $F_{H}≳0.10$ sverdrup up to the tipping point. Last, the surface flux $F_{S}$ (Fig. 3C) onto the North Atlantic for an active AMOC is larger under OBS for lower hosing values, but the roles reverse for larger hosing $F_{H}>0.16$ sverdrup. Together with the freshwater transport from the Arctic, this reversal explains the more saline North Atlantic—and hence stronger AMOC—for low hosing values under CBS and the flip for higher hosing values. The surface forcing during a collapsed AMOC is lower under CBS than under OBS and does not explain the fresher North Atlantic.

Figure 3 (E and F) also displays the other differences between the various freshwater transports into the North Atlantic under CBS and OBS conditions. As discussed, for low hosing values, there is a larger freshwater import under CBS ($\Delta F_{\nabla }>0$), which is caused by a larger import through the southern and zonal boundaries. At the same time, there is a larger export through the northern boundary under CBS ($\Delta F_{\text{north}}>0$). All these differences slowly vanish for increased hosing flux. For the surface flux, we find a larger freshwater input to the ocean’s surface under OBS conditions ($\Delta F_{S}<0$), which reverses for increased hosing. This is mainly due to the difference in the precipitation-minus-evaporation above the North Atlantic ($\Delta F_{P-E}$), while the difference in runoff ($\Delta F_{R}$) is negligible. A closer look (fig. S2A) reveals that this switch in the sign of the *P-E* flux difference occurs because both the difference in rain and snow, as well as in evaporation, switch sign. These, in turn, are mainly related to the reversal in the difference in sea surface temperature (SST) in the North Atlantic, which, in turn, is due to the reversal in the difference of northward heat transport by the AMOC. In other words, the behavior of the *P-E* flux is a result of the heat transported by the AMOC and hence a self-reinforcing mechanism: The stronger AMOC sees a net smaller $F_{P-E}$ flux. Moreover, under CBS, the southward sea ice export from the Arctic is smaller for low hosing, and then this difference also switches sign for increased hosing (fig. S2C). As a consequence, we find the same pattern in the total sea ice area in the North Atlantic, where a larger sea ice area limits evaporation from the ocean’s surface.

To summarize the effect of the Bering Strait closure on the AMOC ON state, it affects the Atlantic-Arctic exchange in two ways. For low hosing values—i.e., a strong AMOC—the southward freshwater and sea ice exports are smaller for a closed Strait than for an open Strait. As a result, the North Atlantic is much more saline (fig. S3A). With the resulting stronger AMOC, we also find a larger total freshwater import, increased SSTs in the North Atlantic, and increased precipitation and evaporation over the North Atlantic. For increased hosing—and so a weaker AMOC—we no longer see a marked difference in the lateral freshwater transports between OBS and CBS, but the sea ice import and area in the North Atlantic are now larger under CBS. This limits evaporation and weakens the AMOC, resulting in lower SSTs and a larger surface freshwater flux. This means that the North Atlantic is now fresher under CBS (fig. S3B) and explains the lower critical hosing value for AMOC tipping. Furthermore, there seems to be a critical hosing level $F_{H,c}\approx 0.161$ sverdrup, beyond which an OBS has a salinifying instead of a freshening effect on the North Atlantic. This corresponds to an equilibrium AMOC strength dropping beneath 16.4 sverdrup, equivalent to a decrease of 16.3% from preindustrial strength.

### CO_2_ forcing

Next, the equilibria of an AMOC ON state under an OBS for various fixed hosing values $F_{H}\in \left[0.00,0.15\right]$ sverdrup are taken and forced by a 1% CO_2_ increase per year (starting at 280 ppm) until a prescribed amount of carbon emissions is reached after which the emission rate is set to zero, following the ZECMIP protocol (*46*). These experiments are repeated, but now, the Bering Strait is immediately closed at the start of the simulation. For both OBS and CBS cases, the safe carbon budget—i.e., the maximum amount of carbon emissions without an AMOC collapse—is determined to within 100 Pg of carbon (PgC).

**Fig. 4.**
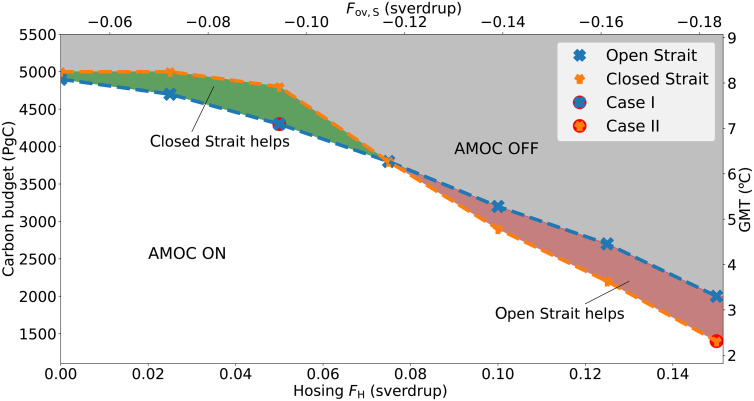
1%/year CO_2_ forcing experiment. The safe carbon budget of the AMOC under OBS and CBS with a starting AMOC state under OBS at various fixed hosing values $F_{H}$. The CO_2_ forcing is increased at a 1% rate until the budget is reached, where either the Bering Strait is kept open (blue marks) or directly closed at the start of the simulation (orange marks). Cases I and II are indicated with an additional red dot. The gray (white) region indicates hosing and carbon budget values under which the AMOC collapses (does not collapse) regardless whether the Strait is closed or open. The green (red) region indicates forcing values under which the AMOC only collapses if the Strait is open (closed). On the right axis, the GMT increase corresponding to the carbon budget emitted is indicated, using 1.65°C/1000 PgC (*47*), and on the top axis, the approximate corresponding $F_{\text{ov},S}$ value of the starting equilibrium state using the linear fit in fig. S5.

The results are shown in Fig. 4. The white and gray regions indicate the amount of carbon emissions and fixed hosing values under which a direct CBS does not alter the outcome. For hosing values $F_{H}\in \left[0.00,0.075\right]$ sverdrup, the safe carbon budgets are higher when the Strait is directly closed at the start of the simulation, while for higher hosing values, the reverse is true and an immediate closure of the Strait would actually reduce the safe carbon budget. The reported emission amounts are related to the corresponding rise in global mean temperature (GMT), using that ~1.65°C/1000 PgC (*47*). Moreover, the hosing values are related to the freshwater transport induced by the overturning circulation in the Atlantic at 35°S ($F_{\text{ov},S}$) in equilibrium, using a least-squares linear fit (see fig. S5). This is done as $F_{\text{ov},S}$ is an important indicator for the strength of the AMOC’s salt-advection feedback and can reveal any biases in the Atlantic’s freshwater budget (*14*, *28*, *48*, *49*). In this model, the computed $F_{\text{ov},S}$ lie neatly within the observed range (*50*).

**Fig. 5.**
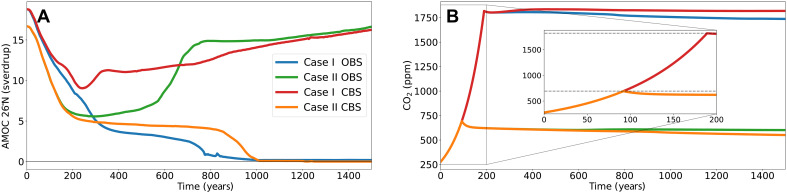
Cases I and II. Case I with a 1%/year CO_2_ increase for 188 years and hosing $F_{H}=0.05$ sverdrup with an open Strait (blue) and an immediate closure (red) and case II with a 1%/year CO_2_ increase for 93 years and hosing $F_{H}=0.15$ sverdrup with an open Strait (green) and an immediate closure (orange), showing the AMOC strength (**A**) and corresponding atmospheric CO_2_ concentration (**B**). The horizontal dashed lines indicate the maximum attained CO_2_ concentration, which is 1820 and 693 ppm for cases I and II, respectively. Note that the CO_2_ concentration drops faster if the AMOC has collapsed, since this affects the marine carbon uptake (*63*).

We will treat two forcing scenarios in more detail (Fig. 4). We consider case I, where the forcing consists of a hosing value of 0.05 sverdrup with a 1%/year increase in CO_2_ for 188 years before emissions are set to zero (leading to a total of 4300 PgC of emissions), and case II with a hosing value of 0.15 sverdrup with a 1%/year increase in CO_2_ for 93 years before emissions are set to zero (leading to a total of 1400 PgC of emissions). In both cases, the initial CO_2_ concentration is 280 ppm. In case I, an immediate CBS prevents an AMOC collapse, while in case II, a collapse occurs only because of the closure (Fig. 5). For all trajectories, the AMOC shows a steep drop in strength under the CO_2_ forcing, with a weakening up to 8.5 sverdrup within the first 300 years. The corresponding CO_2_ concentrations are quite extreme: In case II, the maximum concentration of 693 ppm is reached within a century, and in case I, the maximum of 1820 ppm is attained within two centuries. Under the most extreme shared socioeconomic pathway SSP5-8.5, a global average concentration of 1135.2 ppm in 2100 CE and 2108.3 ppm in 2200 CE is reached (*51*). Within these first 300 years, the recovering trajectories (case I under CBS and case II under OBS) reach their AMOC minima (at 241 and 292 years, respectively), and therefore, the key factor determining whether the AMOC collapses or recovers must already be present in the first three centuries.

**Fig. 6.**
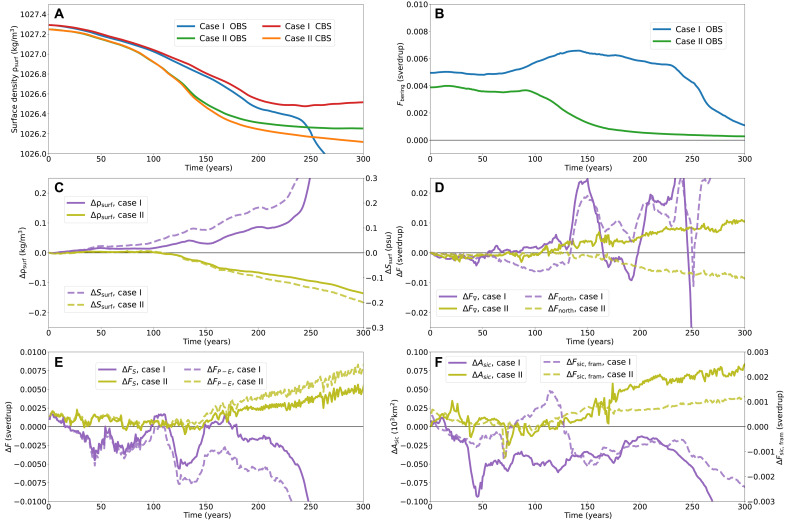
Case I and II diagnostics. Case I with a 1%/year CO_2_ increase for 188 years and hosing $F_{H}=0.05$ sverdrup with an open Strait (blue) and an immediate closure (red) and case II with a 1%/year CO_2_ increase for 93 years and hosing $F_{H}=0.15$ sverdrup with an open Strait (green) and an immediate closure (orange) with their average density $\rho_{\text{surf}}$ of the top 200 m of the North Atlantic region (**A**) and the freshwater transport through the Bering Strait $F_{\text{bering}}$ (**B**). (**C** to **F**) Moreover, the difference between CBS and OBS settings for case I (purple) and case II (citrus) in surface density $\Delta \rho_{\text{surf}}$ (C, solid) and in surface salinity $\Delta S_{\text{surf}}$ (C, dashed), in freshwater import through the lateral boundaries $\Delta F_{\nabla }$ (D, solid) and in freshwater import through the northern boundary $\Delta F_{\text{north}}$ (D, dashed), in surface freshwater transport $\Delta F_{S}$ (E, solid) and in precipitation-minus-evaporation $\Delta F_{P-E}$ (E, dashed), and in sea ice area in the North Atlantic $\Delta A_{\text{sic}}$ (F, solid) and in southward sea ice export through the Fram Strait $\Delta F_{\text{sic},\text{fram}}$ (F, dashed).

In Fig. 6, the surface densities and differences in freshwater fluxes into the North Atlantic region for cases I and II are depicted during the first 300 years of the simulation. Figure S6 depicts the same quantities over the full simulation runtime. Figure 6 (A and C) shows the average surface density in the North Atlantic and the difference in surface density and salinity between the CBS and OBS settings, respectively. Already at the start of the simulations, the density of the collapsing trajectory is lagging behind. Moreover, this coincides mainly with a lag in salinity, and therefore, the cause must be the freshwater inputs into the North Atlantic region. Considering the difference in freshwater import through its lateral boundaries ($\Delta F_{\nabla }$; Fig. 6D), we see that the signal for case II is quite consistent. Here, for most of the first 300 years, there is a higher freshwater import under a closure of the Strait, despite that the freshwater import from the Arctic is lower, i.e., $\Delta F_{\text{north}}<0$.

For case II, we also see a consistently higher surface freshwater forcing under a closure ($\Delta F_{S}>0$; see Fig. 6E), mainly due to a higher precipitation-minus-evaporation over the North Atlantic. Again, we can relate this to a higher sea ice coverage—as this limits evaporation—over the North Atlantic, which is the result of a higher sea ice export from the Arctic to the North Atlantic (see Fig. 6F). Hence, the lowered surface density and salinity under a closure for case II are despite the lowered freshwater import from the Arctic and mainly due to the increased $F_{P-E}$ flux over the North Atlantic, which is related to the increased sea ice export from the Arctic. Note also that, for case II, the freshwater import from the Pacific into the Arctic is actually elevated during the first 250 years despite the weakened AMOC.

Understanding the behavior in case I, where a closure prevents an AMOC collapse, is less straightforward. The difference in the total import of freshwater through the lateral boundaries $\Delta F_{\nabla }$ is quite erratic but mainly higher under CBS for the first 250 years before dropping steeply. The freshwater import from the Arctic, on the other hand, is initially lower under CBS settings for the first 130 years (Fig. 6D). Hence, the initial higher surface density and salinity under a closure are partly due to the reduced freshwater import from the Arctic but only for roughly the first century of the simulation. For the full simulation, the surface freshwater flux is lower under a closure, which is mainly due to a lower precipitation-minus-evaporation (Fig. 6E and fig. S6E). This—again—can be related to the lower sea ice coverage in the North Atlantic with the reduced sea ice export from the Arctic. Hence, overall, the more saline North Atlantic under CBS can be explained by the lower $F_{P-E}$ due to the lower sea ice cover and partly by the reduced freshwater import from the Arctic. Note that, for case II, we see that $F_{\text{bering}}$ reduces more rapidly than in case I, and hence, under OBS, less freshwater is imported to the Arctic from the Pacific in case II than in case I. This agrees with a closure preventing an AMOC collapse in case I but not in case II.

The results of the CO_2_ forcing experiment align partly with those of the hysteresis experiment. For a low hosing value (e.g., $F_{H}=0.05$ sverdrup), a closure results in a more saline North Atlantic and hence a more resilient AMOC, as it limits the freshwater surface forcing via reduced sea ice export from the Arctic and it limits the freshwater export from the Arctic. For a high hosing value (e.g., $F_{H}=0.15$ sverdrup), a closure still reduces the freshwater import from the Arctic, but the increased surface forcing over the North Atlantic—as we also saw for the hysteresis experiment—causes a fresher Atlantic and hence a more vulnerable AMOC. Note that the freshwater import through the Bering Strait under CO_2_ forcing does not align with the results for the freshwater-forced hysteresis experiment, as has also been seen in a previous study (*32*). Despite the AMOC weakening under CO_2_ forcing in case I, this freshwater import actually increases initially. Also, here, it seems that there is a critical hosing value $F_{H,c}$ above which a closure has a net freshening instead of salinifying effect on the North Atlantic. In the case of the CO_2_ forcing used here, we found $F_{H,c}=\left(0.075\pm 0.0125\right)$ sverdrup, which corresponds to an equilibrium AMOC strength of (18.4 ± 0.2) sverdrup under OBS. This is equivalent to a reduction in strength of (6.1 ± 0.5)% from preindustrial. We expect that this $F_{H,c}$ value is dependent on the type and rate of the applied forcing.

### Delay of the closure

**Fig. 7.**
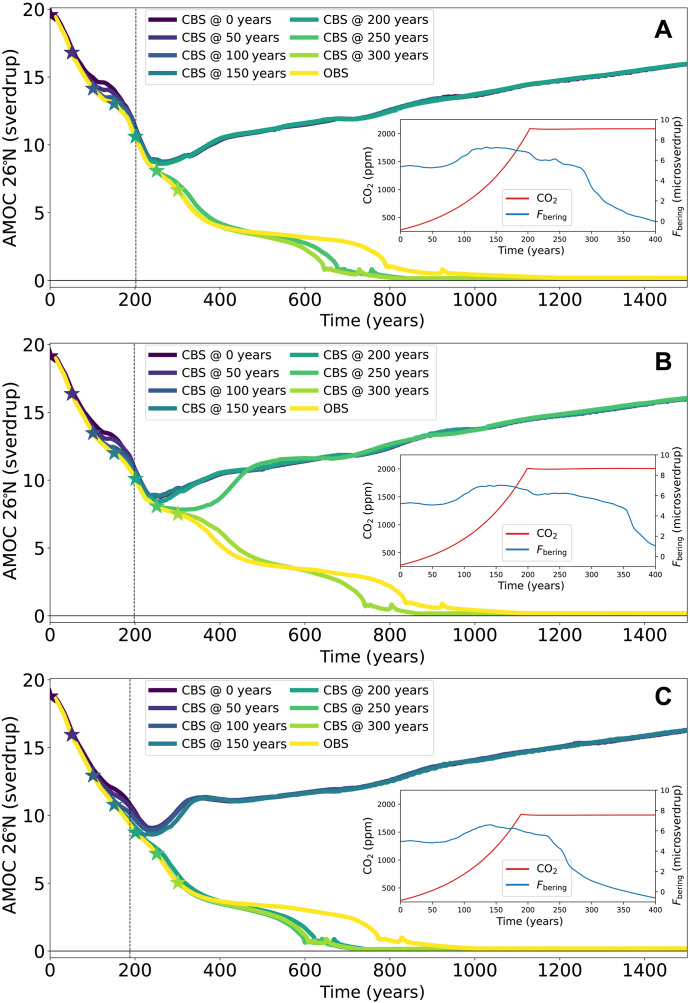
Delay of the closure. The AMOC strength under three forcing scenarios for which an immediate CBS prevents an AMOC collapse: $F_{H}=0.0$ sverdrup with 4900 PgC of emissions (**A**), $F_{H}=0.025$ sverdrup with 4700 PgC of emissions (**B**), and $F_{H}=0.05$ sverdrup with 4300 PgC of emissions (**C**). The closure is done at time 0, 50, 100, 150, 200, 250, and 300 years or not at all (OBS) (purple to yellow, solid). The asterisks in corresponding colors indicate time of closure, and the dashed vertical lines indicate the time when emissions seize. The insets show the corresponding CO_2_ concentrations (red, left axis) and freshwater flux through the Strait (blue, right axis) in the first 400 years for the open Strait scenario.

To study the effect of the timing of the Bering Strait closure, we take the hosing values for which a closure extends the AMOC’s safe carbon budget, and we apply again a 1%/year CO_2_ forcing starting at 280 ppm. This entails the forcing scenarios $F_{H}=0.0$ sverdrup with a CO_2_ increase over 202 years, $F_{H}=0.025$ sverdrup with a CO_2_ increase over 198 years, and $F_{H}=0.05$ sverdrup with a CO_2_ increase over 188 years. This results in a total amount of emissions of 4900, 4700, and 4300 PgC, respectively. They all fall within the previously determined extended safe budget for an immediate closure. However, we now delay a CBS by 50, 100, 150, 200, 250, or 300 years after the forcing has started. The CBS can be delayed up to 200, 250, and 150 years (Fig. 7) for the three forcing scenarios, respectively. On the basis of our previous analyses, we investigate the average salinity of the North Atlantic surface together with the freshwater fluxes through its northern boundary and through its surface as precipitation-minus-evaporation for the first forcing scenario (see Fig. 7A and fig. S7).

First, a closure that is too late to prevent a collapse actually speeds up the AMOC’s collapse. This is again in agreement with the dynamical results from the hysteresis experiment. For low hosing values and correspondingly a sufficiently strong AMOC, a closure inhibits the Arctic’s freshwater and sea ice transport to the North Atlantic and hence strengthens the AMOC. However, if the AMOC is weakened or OFF, then for an OBS, there is a reduction of the freshwater fluxes into the North Atlantic and so a closure would weaken the AMOC. This is the case for a closure after 250 and 300 years in the first forcing scenario, where these belated closures eventually see an elevated *P-E* flux over the North Atlantic and reduced freshwater export to the Arctic from 400 years onward compared to the OBS trajectory. This causes their more rapid collapse, preceding the collapse under OBS by roughly 100 years. These differences in freshwater transports also hasten the freshening of the North Atlantic with the surface salinity at times being up to 2 practical salinity unit (psu) lower for a belated closure compared to an OBS (fig. S7, A to F).

A timely closure, on the other hand, sees directly an increased sea surface salinity in the North Atlantic compared to the OBS. This is mainly due to *P-E*, which is temporarily elevated before dropping below the values of the collapsing trajectories. The freshwater import from the Arctic ($F_{\text{north}}$) is after a closure unexpectedly elevated compared to the OBS base case, although all recovering trajectories see a sharp drop in this import compared to OBS after 300 years. So, for all closures that cause a recovery, we see an immediate increase in surface salinity difference $\Delta S_{\text{surf}}$ following the closure. The same recovery pattern is observed for the belated closure at 250 years, which is halted after several decades since the AMOC has already decreased too much and so the salt-advection feedback has already sufficiently developed. The latest closure at 300 years does not even show this initial recovery, and its surface salinity difference drops shortly thereafter.

This is made more clear by fig. S7 (G to I) where these freshwater fluxes and surface salinity are shown with respect to the AMOC strength. In this phase space, all recovering and all collapsing trajectories seem to converge to roughly one trajectory, respectively. Hence, it seems likely that a preventive closure needs to occur before these two sets of trajectories diverge. As one can see, the latest closure that still results in a recovery (blue asterisk in fig. S7) is also the last closure before the two sets of trajectories separate. Before this divergence, the AMOC is still sufficiently strong and so the immediate strengthening effects of a closure can overcome the developing salt-advection feedback, while a slightly too late closure (at 250 years) still sees an immediate salinifying effect on the North Atlantic but not enough to overcome the salt-advection feedback and prevent a collapse.

Consequently, we can identify critical AMOC strengths below which the salinifying effects of a closure can no longer prevent a collapse for the scenarios presented in Fig. 7 (A to C), which are then 9.4 ± 1.3 sverdrup, 7.8 ± 0.3 sverdrup, and 9.8 ± 1.0 sverdrup, respectively. Note that for the second forcing scenario (Fig. 7B), a preventive closure is still effective for a relatively low AMOC strength. In this scenario, the freshwater transport through the Bering Strait is relatively high for these low strengths, and so, an OBS still has a freshening effect on the Arctic. That this differs with the other scenarios might be related to the fact that the safe carbon budget is determined in increments of 100 PgC, and so in the second scenario, the forcing can be closer to the actual safe carbon budget, and consequently, a lower AMOC is reached without tipping. It is encouraging that there is a large window of opportunity to construct the BSD despite a substantial AMOC weakening having already taken place.

## DISCUSSION

The results presented here indicate that an artificial CBS could be an effective climate intervention strategy to prevent an AMOC collapse under CO_2_ forcing. The hysteresis experiment showed the dynamical effect of a closure on the AMOC and agreed qualitatively with existing literature (*24*–*27*, *29*–*32*). For an active AMOC with a relatively saline North Atlantic, a closure will allow for a reduced freshwater transport out of the Arctic into the North Atlantic and hence cause an AMOC strengthening. For an increasingly fresher North Atlantic and weaker AMOC, an OBS has a stabilizing effect, as it allows for more freshwater to leave the North Atlantic and reduced sea ice import into it. This effect is even more pronounced for a collapsed AMOC. The CO_2_ forcing experiment again showed that a relatively strong AMOC sees a net freshening effect of the Bering Strait, and so, its closure can prevent a CO_2_-induced AMOC tipping, while for a weaker AMOC in the initial equilibrium (i.e., increased hosing value $F_{H}$ beyond the critical hosing $F_{H,c}$), the reverse is true. Last, the closure delay experiment demonstrated that a closure occurring when the AMOC is already severely weakened has a counterproductive effect. However, with a timely closure, its stabilizing effects are able to prevent the development of the salt-advection feedback. Moreover, it also showed an operating window of at least 150 years after the 1% CO_2_ forcing had commenced.

Note that previous studies have found that a CBS would reduce the AMOC’s stability (*27*, *30*, *32*), where, for a sufficiently weak AMOC strength, an OBS allows for additional volume export out of the North Atlantic into the Arctic Ocean. Here, the AMOC is forced by a large freshwater perturbation into the Atlantic, and under OBS, this perturbation can be partially removed to the Arctic and North Pacific, which stabilizes the AMOC. However, we claim in this study that a closed Strait can be stabilizing if the perturbation to the AMOC is by climate forcing, i.e., induced by a temperature increase rather than a salinity decrease of the upper Atlantic. In this case, this stabilizing effect of an open Strait is limited, while the open Strait still allows for the entry of additional sea ice from the Arctic. Once we have a weaker AMOC as a starting state with a corresponding higher freshwater content in the North Atlantic, this stabilizing mechanism of an OBS is more effective in combination with a now higher sea ice coverage under closure. So, in this latter case, a closure becomes again a destabilizing effect.

The technical feasibility of the BSD is supported by the fact that its construction challenges are on par with already completed mega-projects. As stated earlier, the BSD would have a total length of roughly 80 km, with an average and maximum depth of 50 and 59 m, respectively. By way of comparison, the current largest enclosure dam is the Saemangeum Seawall (South Korea) with a length of 33 km and a maximum depth of 54 m (*41*). So, in both dimensions, the BSD would be of the same order of magnitude. Moreover, if we assume the BSD to rise 20 m above sea level and to be 100 m wide at the top with two sloping sides of 1:2 (height:width) ratio (*52*), then roughly 1.3 km^3^ of raw material is needed to build the dam. This is only a factor 3.5 larger than the amount needed to construct Maasvlakte 2, the extension of the Port of Rotterdam (*41*). Hence, also in this aspect, the required dimensions stay within the same order of magnitude as already developed projects. A more detailed feasibility analysis is beyond the scope of this study.

Although CLIMBER-X is ideally suited to study the detailed mechanisms of the AMOC’s response to various forcings (*42*, *44*, *45*)—including a Bering Strait closure under climate change—its horizontal spatial resolution of 5° × 5° is very coarse. The BST is modeled as a baroclinic tracer exchange between the Arctic and North Pacific, while in reality, it is a mainly barotropic flow that is geostrophically balanced by the sea surface height difference between the Arctic and North Pacific Ocean. Hence, the model does only provide the freshwater and heat exchange through the Strait. As already mentioned above, this gives discrepancies with observations on volume and freshwater transport values (*22*). However, qualitatively, the dynamics in CLIMBER-X coincide with existing studies, some done at higher resolutions (*24*, *27*, *30*, *31*), and so, we are confident in the results and the generality of the physical mechanisms. We expect a reproduction of our experiments with a more detailed model that does directly resolve the Strait—if computationally feasible—will only affect our results quantitatively.

Another limitation is in the choice of the applied forcings. This hosing was varied to explore the response of various AMOC ON states with different freshwater budgets and strengths. This is done as the freshwater budget of the preindustrial AMOC has slight biases in the model (*42*, *49*). As a consequence, however, the freshwater hosing does become unrealistically high up to 0.15 sverdrup, which is roughly a factor 20 larger than the present-day melt rate of the Greenland Ice Sheet (GIS) (*53*). The climate forcing consisted of solely the CO_2_ concentration being increased at a 1% rate per year. This was done instead of a SSP scenario for the sake of simplicity albeit less realistic (*54*). To cause an AMOC collapse, total carbon emissions of up to 5000 PgC were needed. Although large, this amount still falls below the upper estimate of available fossil fuel reserves (*55*), and the corresponding CO_2_ concentrations fall below those computed for the extended SSP5-8.5 scenario as well (*51*).

All in all, because of the limitations of both the forcing scenarios and of the model, the results presented in this study are mainly conceptual. They indicate that there are forcing scenarios for which a closure prevents an AMOC collapse, although the window of climate and freshwater forcings for which this holds is rather limited in CLIMBER-X. The relevant range of freshwater and CO_2_ forcings for which the BSD is effective is only 0.075 sverdrup and 500 PgC wide at best—small compared to the critical threshold values of 0.22 sverdrup and 5000 PgC of a preindustrial AMOC. However, compared to observed maximum freshwater flux of the GIS (0.015 sverdrup) and the total cumulative global fossil fuels emissions up to 2024 (505 PgC), this range is relatively large (*53*, *56*), and so solely based on our adopted model’s result, it is unlikely to enter or leave this effective range readily. Moreover, we expect that this extent is larger in more detailed models than presented in this study, as the effect of a closure on the AMOC strength and the total freshwater transport through the Strait under preindustrial conditions is an order of magnitude lower in our adopted model than in more detailed studies (*21*, *24*). Repeating the closure experiments in a more detailed climate model under more realistic climate forcings will allow us to make a more quantitative assessment on whether a CBS is able to prevent an AMOC collapse in our present-day climate. This will yield a more accurate quantitative assessment of the BSD’s effect and can help determine the extent of the forcing scenarios for which a closure prevents a collapse. In addition, as it is qualitatively now expected that there is a critical hosing value $F_{H,c}$ (and corresponding equilibrium AMOC strength) below (above) which a closure aides the AMOC’s resilience to this climate forcing, additional simulations are needed to improve the estimate of this value and relate it to current observations. Hence, our results are a strong indication that a BSD can be a feasible climate intervention strategy and invite further exploration.

A next step could be to simulate a closure in the Community Earth System Model: a state-of-the-art climate model in which an AMOC collapse has been found under RCP4.5 forcing scenario with a more accurate Atlantic freshwater budget (*10*, *15*). Last, for a full consideration of the BSD as an alternative climate intervention, analyses regarding its technical feasibility, economic effects, and environmental impacts are needed. We expect the BSD to have a large impact onto the regional ecosystem (*57*), and so particularly in this regard, we do want to stress that CO_2_ mitigation efforts are the preferable option to avoid an AMOC collapse. But if this is not realized, this study showed that in an Earth system Model of Intermediate Complexity (EMIC), a man-made timely CBS can prevent a collapse of the AMOC under particular climate forcing scenarios.

## MATERIALS AND METHODS

### CLIMBER-X

CLIMBER-X (*42*, *43*) is a fast EMIC using the frictional geostrophic three-dimensional (3D) ocean model GOLDSTEIN (*58*) together with the semiempirical statistical-dynamical atmospheric model SESAM (Semi-Empirical dynamical Statistical Atmosphere Model) (*42*), a dynamic-thermodynamic sea ice model SISIM (Simple Sea Ice Model) (*42*), and the land surface model with interactive vegetation PALADYN (*59*). There are also components for ocean biogeochemistry (HAMOCC) and ice sheets (SICOPOLIS or Yelmo), but these are not used in this study. Note that, therefore, ice sheets are prescribed at their modern state, and so, the net freshwater flux from these sheets is assumed to be zero. Hence, in our simulations with climate forcing, the AMOC is not affected by increased melt from, e.g., the GIS. As mentioned before, all components of the climate model have a horizontal resolution of 5° × 5°.

The atmosphere model SESAM uses a combination of observational data as well as results from global climate models where all prognostic variables (e.g., temperature, humidity, and wind speed) are determined on a 2D grid, while the vertical structure is purely diagnostic. The general vertical structure in the atmosphere of humidity and temperature is used to determine the complete 3D structure of these variables, while the thermal wind balance is used to compute the 3D structure of the wind. Longwave radiation fluxes take into account several greenhouse gases such as methane, chlorofluorocarbons (CFCs), ozone, and CO_2_. Clouds are also represented with one cloud layer which is characterized by variables such as cloud fraction and albedo.

The ocean model GOLDSTEIN is run on 23 nonequidistant vertical layers. Horizontal velocities are derived from a frictional-geostrophic balance, while vertical velocities follow from the continuity equation. Throughout the water column, a hydrostatic balance is assumed. Moreover, a rigid-lid approximation is assumed, and therefore, surface freshwater fluxes are represented as virtual salinity fluxes. Note that the 5° × 5° rectilinear grid is too coarse to represent the Bering Strait directly: The Strait is enclosed in the grid cell centered at 67.5°N and 167.5°W, which has an ocean fraction of 0.84. An open Strait is modeled by allowing baroclinic tracer exchange between the Arctic and North Pacific Ocean, while the Strait is always closed for barotropic flow. A closure of the Strait entails a seizing of the tracer exchange. Consequently, the Throughflow’s strength in this model is not realistic, while its effect on the buoyancy of the North Atlantic is, as the exchange of tracers such as freshwater and temperature is captured. The model’s performance is comparable with state-of-the-art Coupled Model Intercomparison Project Phase 6 (CMIP6) models under various forcings and boundary conditions. In particular, the deep convection zones in the model coincide with those following ocean reanalysis, while the AMOC’s overturning pattern at 26°N is quite similar to the RAPID observations, although the modeled AMOC is a bit too shallow. Moreover, because of the strong momentum damping, the Antarctic Circumpolar Current is too weak.

The sea ice model SISIM models sea ice as one snow layer on top of one ice layer. The snow can accumulate and melt, and if it exceeds 1 m, then the excess becomes ice. This ice layer cannot only accumulate from above but also grow via accretion from below and melt from above and from below. The freezing temperature is dependent on the local ocean salinity. Moreover, the sea ice is allowed to drift. Last, SISIM also acts as an ocean-atmosphere coupler even in sea ice–free regions.

The land module PALADYN computes the water and energy fluxes between the land surface, soil, and atmosphere. It represents the terrestrial carbon cycle including dynamical vegetation. Water, temperature, and carbon contents are solved in the soil using five vertical layers.

### Climate model simulations

The hysteresis experiment is performed with a prescribed freshwater flux $F_{H}$ into the Atlantic latitudinal belt 20°N to 50°N. This freshwater hosing is compensated globally. The first hysteresis simulation starts in the preindustrial equilibrium, i.e., no hosing and with CO_2_ at 280 ppm, after which the hosing is increased at a rate of 0.025 sverdrup/kyr till the total hosing is 0.35 sverdrup. Then, it is reduced with a rate of −0.025 sverdrup/kyr till the total hosing is −0.25 sverdrup, and last, it is again increased with the same rate back to zero hosing. The total simulation takes 48,000 years. For the second hysteresis, we repeat this protocol, but we start in the equilibrium with a closed Bering Strait and active AMOC under preindustrial conditions. This state is computed with a 7000-year-long simulation that starts in the original preindustrial equilibrium where the Strait is directly closed at start. During the hysteresis experiment, the applied freshwater forcing is compensated by removing freshwater from the surface ocean globally to prevent a drift in the salinity field. This allows us to use the states of the hysteresis experiment as starting states for the subsequent experiments, where the goal was to have various starting states with an active AMOC with the same total salinity but differing strengths of the corresponding salt-advection feedback. This strength is demonstrated by the freshwater transport by the overturning circulation at 34°S $F_{\text{ov},S}$ (see fig. S5) (*10*, *28*). Previous studies indicate that changing the compensation scheme only affects the location of the AMOC’s tipping point (*60*, *61*).

For the 1% CO_2_ forcing experiment, the starting states for fixed freshwater fluxes with an open Strait are taken from the hysteresis simulation. The CO_2_ concentration is increased at 1%/year (starting at 280 ppm) until the prescribed emission budget is reached after which no emissions occur, following the ZECMIP protocol (*46*). The simulations run for 1500 years. At the start of the simulation, the Strait is either closed or kept open. The safe budget is found by increasing the emissions with increments of 100 PgC until the AMOC collapses.

For the third experiment, this setup is repeated for the mentioned forcing scenarios, but now, the closure is delayed up to 300 years in increments of 50 years after the simulation start.

### The AMOC strength

The AMOC strength is defined as the maximum of the Atlantic meridional overturning streamfunction $\psi_{A}\left(y,z\right)$ at 26°N

$$\text{AMOC}={\text{max}}_{y=26^{\circ }N}\left[\psi_{A}\left(y,z\right)\right]$$ (1)

where the streamfunction is computed as

$$\psi_{A}\left(y,z\right)=-\int_{z}^{0}\int_{x_{W}\left(y,z\right)}^{x_{E}\left(y,z\right)}\;v\left(x^{\prime },y,z^{\prime }\right)\;dx^{\prime }\;dz^{\prime }$$ (2)

where *v* is the meridional velocity, and $x_{W}$ and $x_{E}$ are the western and eastern boundary of the Atlantic basin, respectively.

### AMOC tipping point estimate

The tipping points of an AMOC collapse based on the hysteresis simulations in Fig. 2 are determined as the last point before the collapse where it still holds that $\frac{\partial \text{AMOC}}{\partial F_{H}}>-1$. During an AMOC collapse, we have $\frac{\partial \text{AMOC}}{\partial F_{H}}<-1$: The changes in AMOC strength are then primarily driven by internal feedbacks instead of changes in the external forcing.

### Freshwater transports into the North Atlantic region

The region we consider the North Atlantic is the region in the Atlantic between the latitudes 50°N and 75°N and longitudes 75°W and 55°E (see fig. S8). This region is chosen as it encompasses most of the mixed layer zones in the model. The surface density (or salinity) is computed as the average density (or salinity) of the top 200-m layer in this region. We compute four freshwater transports, one for each boundary section. A positive sign indicates a net import into this region. These are computed as

$${\left.F_{\text{north}}=-\int_{-H}^{0}\int_{75^{\circ }W}^{55^{\circ }E}\;v\left(1-\frac{S}{S_{0}}\right)\right|}_{75^{\circ }N}\;\mathrm{dx}\;\mathrm{dz}$$ (3A)

$${\left.F_{\text{east}}=-\int_{-H}^{0}\int_{50^{\circ }N}^{75^{\circ }N}\;u\left(1-\frac{S}{S_{0}}\right)\right|}_{55^{\circ }E}\;\mathrm{dy}\;\mathrm{dz}$$ (3B)

$${\left.F_{\text{south}}=\int_{-H}^{0}\int_{75^{\circ }W}^{55^{\circ }E}\;v\left(1-\frac{S}{S_{0}}\right)\right|}_{50^{\circ }N}\;\mathrm{dx}\;\mathrm{dz}$$ (3C)

$${\left.F_{\text{west}}=\int_{-H}^{0}\int_{50^{\circ }N}^{75^{\circ }N}\;u\left(1-\frac{S}{S_{0}}\right)\right|}_{75^{\circ }W}\;\mathrm{dy}\;\mathrm{dz}$$ (3D)

where *u* and *v* are the zonal and meridional velocity, respectively, $S_{0}=34.7$ psu is the reference salinity, and *H* is the local water depth. Then, the net freshwater import through the zonal boundaries of the North Atlantic ($F_{\text{zonal}}$) and the net import through all lateral boundaries ($F_{\nabla }$) can be computed

$$F_{\text{zonal}}=F_{\text{east}}+F_{\text{west}}$$ (4A)

$$F_{\nabla }=F_{\text{north}}+F_{\text{east}}+F_{\text{south}}+F_{\text{west}}$$ (4B)

The freshwater transport through the Bering Strait is similarly computed as the meridional freshwater transport through the 65th parallel north between the Pacific and Arctic Ocean.

We also consider the freshwater flux $F_{S}$ through the North Atlantic’s surface, following

$$F_{S}=F_{H}+F_{R}+F_{P-E}+F_{\text{rest}}$$ (5)

where $F_{H}$ represents the hosing flux, $F_{R}$ runoff (e.g., river outflow), $F_{P-E}$ the precipitation-minus-evaporation (e.g., rain, snow, and evaporation), and $F_{\text{rest}}$ a minor rest term to close the budget consisting of calving, brine rejection, and other processes. Moreover, for the precipitation-minus-evaporation, it holds that

$$F_{P-E}=F_{\text{rain}}+F_{\text{snow}}+F_{E}$$ (6)

with rainfall $F_{\text{rain}}$, snowfall $F_{\text{snow}}$, and evaporation $F_{E}$. Note that we have omitted the hosing $F_{H}$ here. For the last term, we have that

$$F_{E}=-\left[f_{\text{sic}}F_{E,\text{sic}}+(1-f_{\text{sic}})F_{E,\text{ocn}}\right]$$ (7)

where $f_{\text{sic}}$ is the fraction of surface that is covered by sea ice, and $F_{E,\text{sic}}$ indicates evaporation via sublimation of sea ice while $F_{E,\text{ocn}}$ indicates direct evaporation from the sea surface. As the former process is not as effective as the latter, evaporation is limited by sea ice cover.

### Software and model output

The (processed) model output and analysis script are provided at https://doi.org/10.5281/zenodo.17640379 and https://doi.org/10.5281/zenodo.17643008. CLIMBER-X is an open-source fast Earth system model and can be found at https://github.com/cxesmc/climber-x.
