# Supplementary material for: The effects of a constructed closure of the Bering Strait on AMOC tipping behavior
Source: Sci Adv. 2026 Apr 24;12(17):eaeb7887. doi: 10.1126/sciadv.aeb7887 (PMC13108545; doi:10.1126/sciadv.aeb7887)
Supplement: Supplementary file 1 — Supplementary text Figs. S1 to S8 [file sciadv.aeb7887_sm.pdf]

Science Advances
AAAS

Supplementary Materials for

The effects of a constructed closure of the Bering Strait on AMOC tipping behavior

Jelle Soons and Henk A. Dijkstra

Corresponding author: Jelle Soons, j.soons@uu.nl

Sci. Adv. **12**, eaeb7887 (2026)
DOI: 10.1126/sciadv.aeb7887

This PDF file includes:

Supplementary text
Figs. S1 to S8

Freshwater forcing for backward hysteresis simulations

Regarding the quasi-equilibrium simulations for decreasing hosing (i.e. with mainly a collapsed AMOC), we see a more severe difference in freshwater exchanges than for the forward simulations, see also Figure S4. For a collapsed AMOC with a closure there is a much larger freshwater transport southward out off the Arctic, resulting in a much fresher North Atlantic, see Figure S3**C**. Note however, that the North Pacific on the other hand is much more saline. With these larges discrepancies in sea surface salinities we also observe large differences in precipitation, evaporation and sea ice area between the OBS and CBS settings. In surface forcing to the North Atlantic the open Strait scenario exceeds that of a closed Strait, while the opposite is true for sea-ice export to it, see Figure S2**B&D**. Hence the much fresher sea surface under CBS must be caused by the larger southward freshwater and sea-ice transport from the Arctic.

Supplementary figures

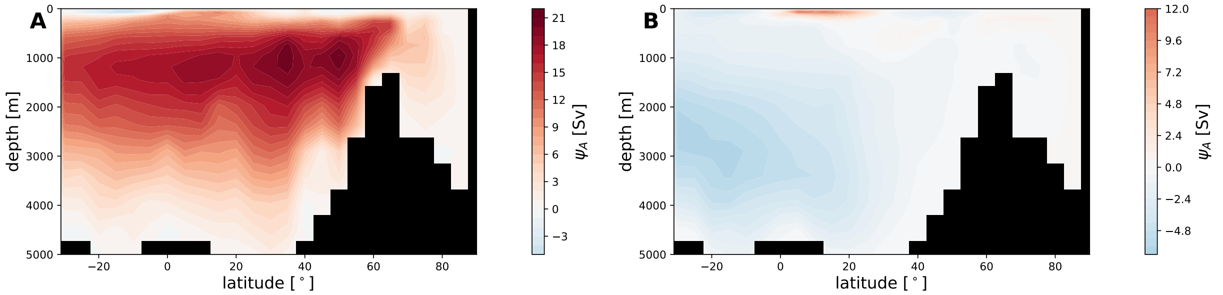

Figure S1: **The Atlantic overturning circulation.** The two equilibria of the AMOC at hosing $F_H = 0.1$ Sv and fixed CO_2 at 280 ppm, with the overturning streamfunction ψ_A depicted in the Atlantic basin of an ON-state (**A**) and an OFF-state (**B**).

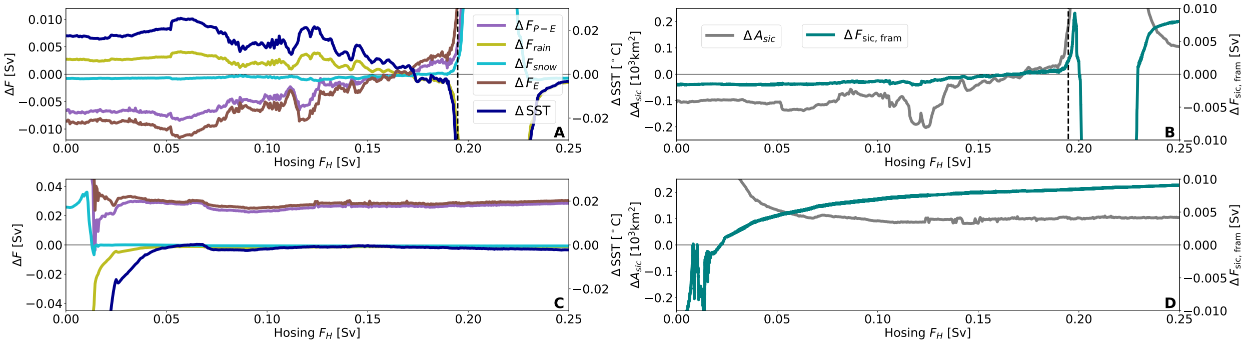

Figure S2: **Precipitation-minus-evaporation and sea-ice to the North Atlantic.** The difference between CBS and OBS conditions for the precipitation-minus-evaporation ($\Delta\, F_{P-E}$, purple), and its components rain ($\Delta\, F_{\text{rain}}$, citrus), snow ($\Delta\, F_{\text{snow}}$, purple), and evaporation ($\Delta\, F_E$, cyan) on the left axis, and for the average sea surface temperature (SST, blue) of the North Atlantic on the right axis, for the quasi-equilibrium simulations with (**A**) an active AMOC, and (**C**) a collapsed AMOC. The difference between CBS and OBS conditions for the sea ice are in the North Atlantic ($\Delta\, A_{\text{sic}}$, gray) on the left axis, and for the southward sea ice export through the Fram Strait ($\Delta\, F_{\text{sic, fram}}$, teal) on the right axis, for the quasi-equilibrium simulations with (**B**) an active AMOC, and (**D**) a collapsed AMOC. The vertical dashed lines indicate the AMOC tipping point under CBS.

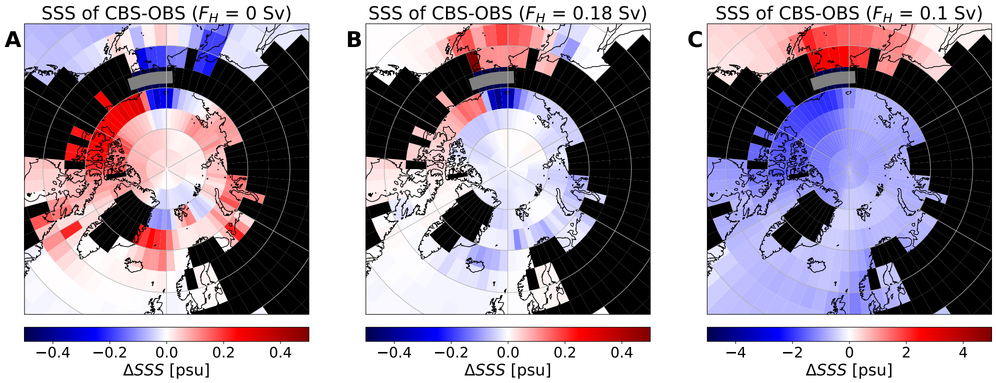

Figure S3: **Difference in sea surface salinities in the Arctic and North Atlantic.** The difference in sea surface salinities (SSS) in the Arctic and North Atlantic north of 55°N between CBS and OBS for the equilibria AMOC ON states at $F_H = 0$ Sv (**A**) and at $F_H = 0.18$ Sv (**B**), and for the equilibria OFF states at $F_H = 0.1$ Sv (**C**). The gray cells represent the BSD.

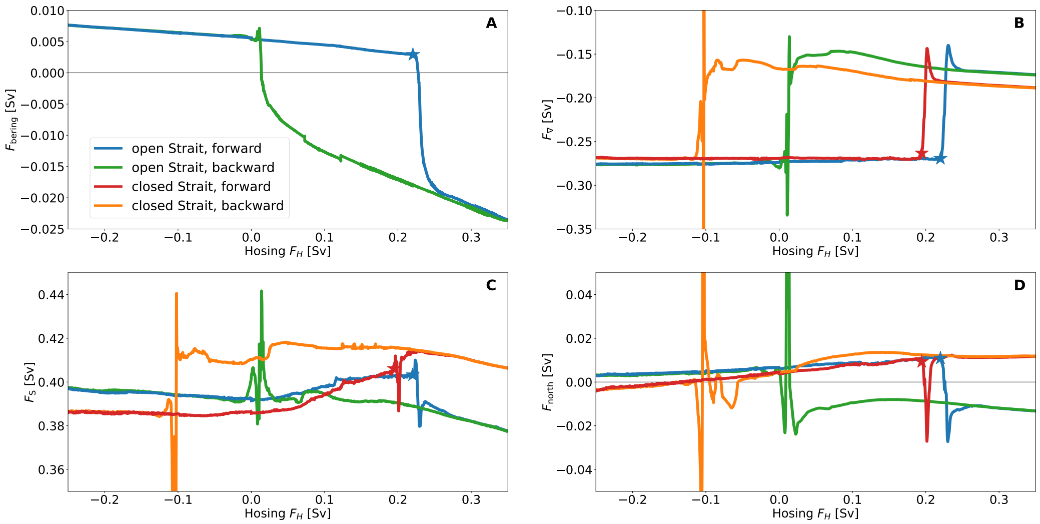

Figure S4: **The freshwater transports for complete hysteresis experiment.** Freshwater transports for the quasi-equilibrium simulations for an open Strait (blue, green), and a closed Strait (red, orange), consisting of simulations where the hosing flux F_H increases (blue, red) and decreases (green, orange) (**A-D**). The asterisks mark the estimated tipping points of the AMOC collapses (**A-D**). (**A-D**) The freshwater transports through, respectively, the Bering Strait (F_{bering}), the lateral boundaries of the North Atlantic region (F_{∇}), the surface of the North Atlantic region (F_S), and the northern boundary of the North Atlantic region (F_{north}).

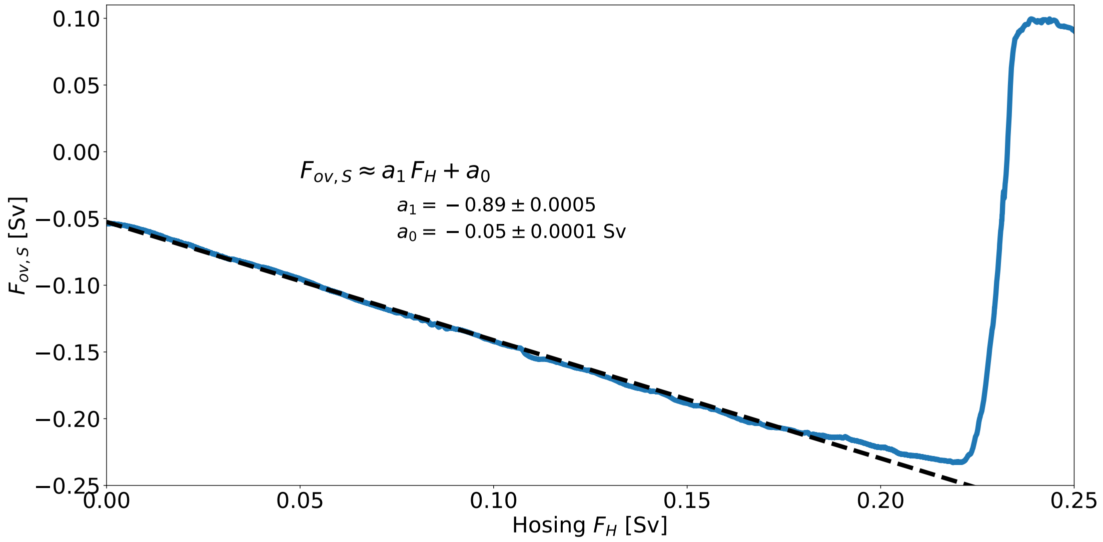

Figure S5: **Linear fit of $F_{ov,S}$ to the hosing.** The $F_{ov,S}$ (blue) of the quasi-equilibrium simulation starting in the AMOC ON equilibrium for OBS with hosing flux F_H increasing at a rate 0.025 Sv/kyr, together with a linear least-squares fit (black, dashed) to the data for range $F_H \in [0.00, 0.20]$ Sv.

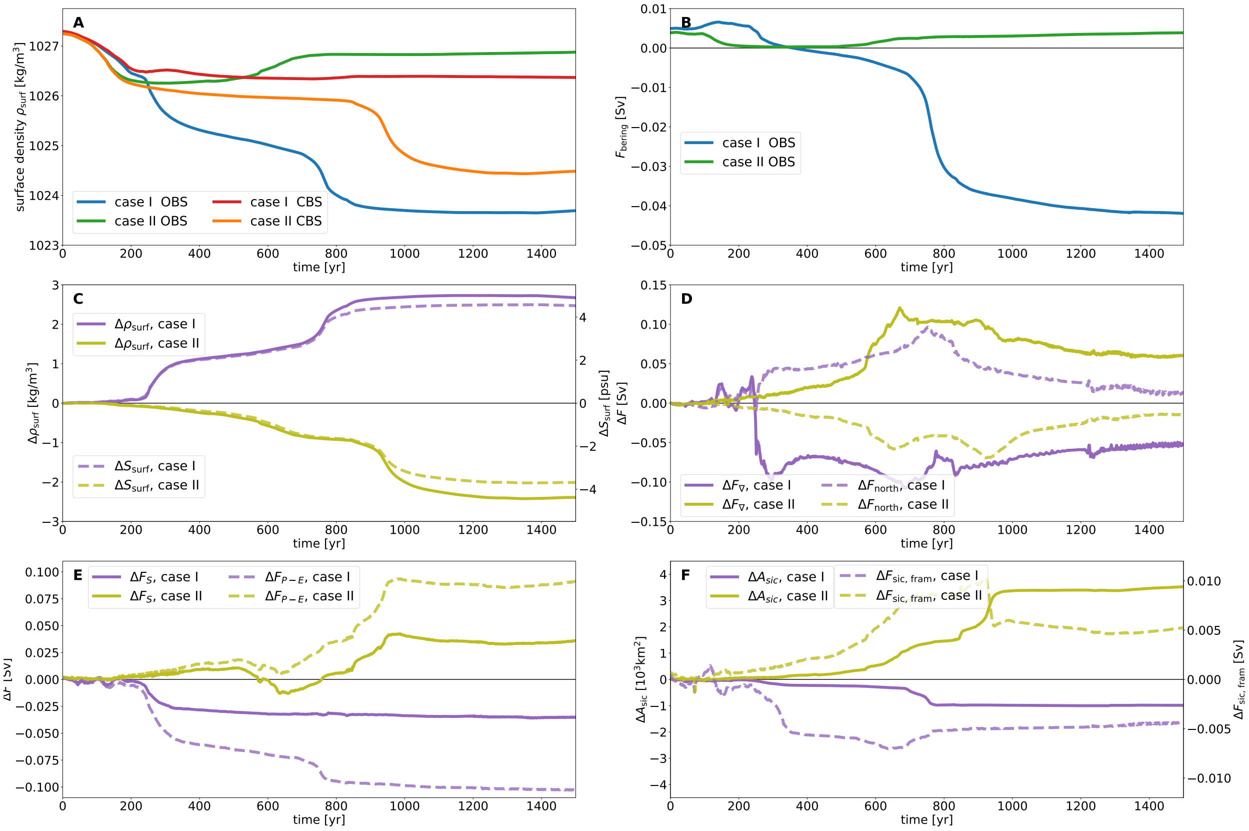

Figure S6: **Case I & II diagnostics for complete simulation runtime.** Case I with a 1 %/yr CO_2 increase for 188 yr and hosing $F_H = 0.05$ Sv with an open Strait (blue) and an immediate closure (red), and Case II with a 1 %/yr CO_2 increase for 93 yr and hosing $F_H = 0.15$ Sv with an open Strait (green) and an immediate closure (orange) with their average density ρ_{surf} of the top 200 m of the North Atlantic region (**A**), and the freshwater transport through the Bering Strait F_{bering} (**B**). Moreover, the difference between CBS and OBS settings for case I (purple) and case II (citrus) in surface density $\Delta\rho_{\text{surf}}$ (**C**, solid) and in surface salinity ΔS_{surf} (**C**, dashed), in freshwater import through the lateral boundaries ΔF_∇ (**D**, solid) and in freshwater import through the northern boundary ΔF_{north} (**D**, dashed), in surface freshwater transport ΔF_S (**E**, solid) and in precipitation-minus-evaporation ΔF_{P-E} (**E**, dashed), and in sea-ice area in the North Atlantic ΔA_{sic} (**F**, solid) and in southward sea-ice export through the Fram Strait $\Delta F_{\text{sic, fram}}$ (**F**, dashed).

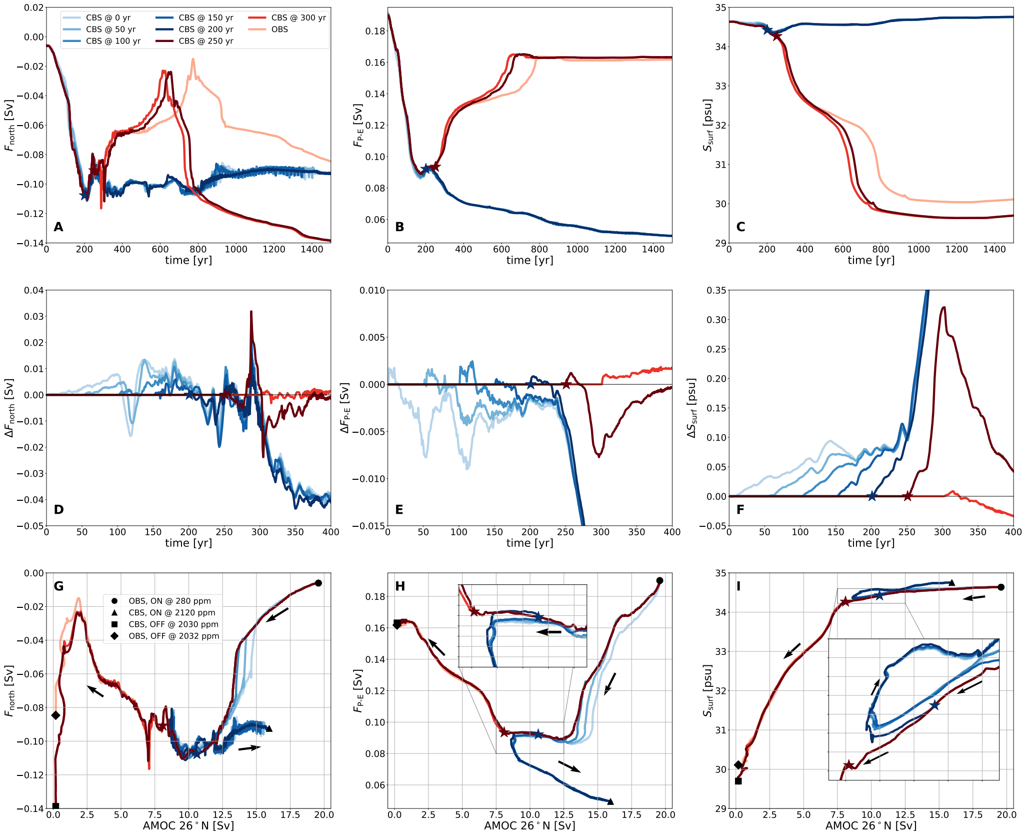

Figure S7: **Diagnostics for delayed closure.** Under forcing scenario $F_H = 0.0$ Sv with 4900 PgC of emissions the Bering Strait is closed at either 0 yr, 50 yr, 100 yr, 150 yr, or 200 yr (light to dark blue, solid) preventing an AMOC collapse, or at 250 yr, 300 yr or not at all (OBS) (dark to light red, solid) with a collapsing AMOC. Depicted are the freshwater import through the northern boundary of the North Atlantic (F_{north}) versus time (**A**), the precipitation-minus-evaporation onto the North Atlantic ($F_{\text{P-E}}$) versus time (**B**), the average surface salinity (S_{surf}) of the North Atlantic versus time (**C**), and the respective differences with the OBS trajectory (ΔF_{north}, $\Delta F_{\text{P-E}}$ and $S_{\Delta\text{surf}}$, **D**, **E** and **F**). The same trajectories are shown in the parameter spaces of F_{north} versus AMOC strength (**G**), $F_{\text{P-E}}$ versus AMOC strength (**H**) and S_{surf} versus AMOC strength (**I**), where arrows indicate the direction of time and markers indicate the starting state and various end states. The blue (red) asterisk marks the latest (earliest) possible closure for which the AMOC recovers (collapses).

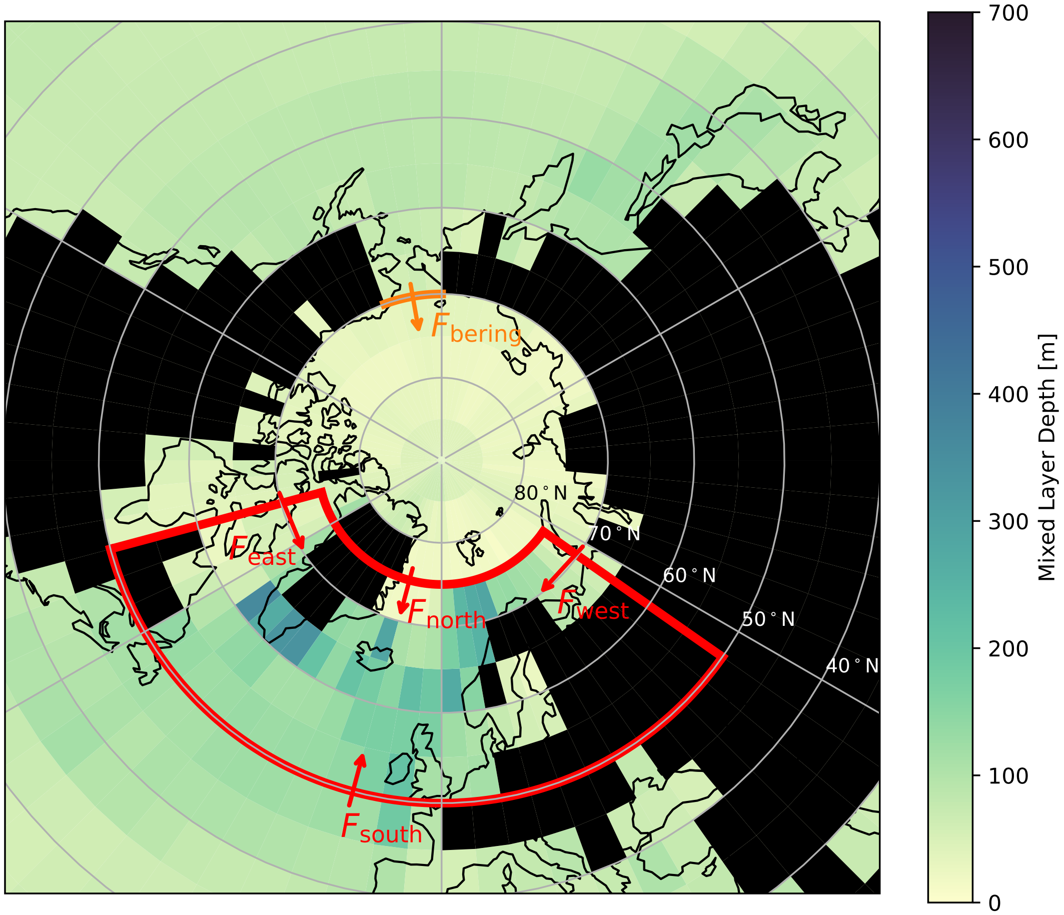

Figure S8: **The North Atlantic.** The selected North Atlantic region (enclosed in red) between latitudes 50°N and 75°N, and longitudes 75°W and 55°E, with the arrows indicating the direction of the computed freshwater transports through each boundary. The orange line indicates the section through which F_{bering} is computed, with the arrow indicating its direction. The yearly-average mixed layer depth of an active AMOC for pre-industrial settings is shown. The black cells indicate grid cells with a zero ocean fraction, on top of the current coastlines (black, solid).
